# Linking Polymer Transformation and Soil Microclimate to Mulch (Bio)Degradation: A Field-Based Approach Using Mesh Bags and FTIR

**DOI:** 10.3390/molecules31101758

**Published:** 2026-05-20

**Authors:** Corina Carranca, Filipe Pedra, Gustavo Brunetto, Joana Barata

**Affiliations:** 1 INIAV—National Institute for Agricultural and Veterinary Research, Qta. Marquês, Av. República, Nova Oeiras, 2784-505 Oeiras, Portugal; filipe.pedra@iniav.pt; 2 Green-It—Bioresources for Sustainablity, ITQB NOVA, 2780-157 Oeiras, Portugal; 3 Soil Department, Universidade Federal Santa Maria, Rio Grande do Sul 97105-900, Brazil; brunetto.gustavo@gmail.com; 4 CESAM—Centro de Estudos do Ambiente e do Mar, Edifício 3, Piso 5, Campus Universitário de Santiago, Universidade de Aveiro, 3810-193 Aveiro, Portugal; jbarata@ua.pt

**Keywords:** ATR-FTIR spectroscopy, biodegradable mulches, conventional plastic mulches, in situ mass loss, Mediterranean conditions, polymer (bio)degradation, soil microclimate

## Abstract

The degradation of mulch materials in perennial cropping systems is governed by both polymer properties and environmental conditions. Their relative influence under field conditions remains unclear. To our knowledge, this study is one of the first to integrate mass loss measurements, polymer characterization, and soil microclimatic assessment under field conditions. A one-year field experiment was conducted under irrigated Mediterranean conditions to compare the degradation of Kraft^®^ paper and polybutylene adipate terephthalate (PBAT)-based (Kritifil^®^) mulch with polypropylene (PP) geotextile fabric and polyethylene (PE) mulch in randomized blocks, with three replicates. Mass loss was quantified in situ using mesh bags, while soil moisture, temperature, and electrical conductivity (EC) were monitored monthly to characterize microclimatic and edaphic conditions underlying mulch treatments. Polymer changes were assessed by ATR-FTIR analysis of field-exposed mulch fragments. Kraft^®^ paper degraded rapidly (≈72% mass loss), consistent with moisture-driven biological processes and susceptibility to hydrolysis. In contrast, PBAT-based mulch showed limited degradation (≈3.5%) despite favourable conditions, suggesting constraints in enzymatic activity. No mass loss was observed for PE- and PP-based mulch. ATR-FTIR analysis indicated minimal structural changes in PBAT, PP, and PE, reflecting their high stability. Overall, polymer composition and inherent (bio)degradability, rather than soil thermal time, were the main drivers of mulch (bio)degradation under Mediterranean conditions.

## 1. Introduction

The global use of agricultural plastic films for food production is estimated to increase by approximately 60% since 2018 [1], with annual consumption potentially exceeding two million tons in Asia and Europe, and particularly in the Mediterranean region [2]. In agricultural systems, mulches play a key role in regulating soil temperature, reducing soil water evaporation, protecting crops from weed competition, and pests and diseases. However, plastic mulches manufactured from fossil-derived polymers, such as polyethylene (PE) and polypropylene (PP), pose a growing environmental concern due to their persistence and long-term accumulation in soils. In fact, the stability of PE plastic film [1,2] usually exceeds the annual crop growth cycles and cannot always be completely removed from the field after harvest, leading to the accumulation of residues in the soil, which may negatively affect soil health and plant growth. In contrast to annual cropping systems, in perennial crops such as blueberry, mulches are expected to remain in place for several years, depending on farmers’ objectives. Consequently, the chemical and physical properties of mulches designed for perennial production systems are expected to differ substantially from those used in annual systems. Mulches derived from organic materials such as wood chips, sawdust, or pine bark, have long been used in commercial perennial fruit production. In contrast, as plastic mulches are a relatively recent innovation, their agronomic performance and ecological effects are not yet understood [3].

The adoption of alternative mulch materials, including bio-based and biodegradable mulches, represents a promising strategy to mitigate the environmental impacts and disposal challenges associated with plastic residues in agriculture. According to the European Standard [4], a mulch film is considered biodegradable when it is degraded and assimilated by soil organisms up to approximately 90% of the material within two years under aerobic conditions and constant temperature (20–28 °C), resulting in its conversion into CO_2_, water, and microbial biomass or inorganic N compounds. But biodegradable mulches must be manufactured from 100% bio-based materials (no fossil-derived materials or synthetic polymers), and also without the use of genetically modified organisms including feedstock and processing microorganisms [5]. Currently, several biodegradable mulch films are commercially available, while additional formulations remain under development and testing. These commercially available films, certified as biodegradable in soil, should be specifically designed to be incorporated into the soil at the end of the cropping season, where they are intended to undergo complete biodegradation by soil microorganisms [6]. Despite these advantages, the adoption of biodegradable mulch films by farmers has been relatively slow, largely due to perceptions of higher costs and concerns regarding premature or inconsistent degradation during the cropping season, and concerns about the environmental fate of polymers [5,7,8]. In fact, there is a limited understanding of the degradation dynamics of these mulch films under different environmental conditions, particularly biodegradable formulations [1,7].

The (bio)degradation rates of mulch films, whether conventional plastic or biodegradable, depend not only on their chemical composition but also on a range of external factors [6,9]. Abiotic drivers, including climatic conditions (wind, UV radiation, air temperature, rainfall, and relative humidity), soil type, crop species, and management practices, strongly influence degradation processes. In addition, biotic factors, primarily fungi and bacteria, as well as actinomycetes and algae, play a key role in the breakdown of these materials. Graf et al. [9] reported that the continuous incorporation of biodegradable mulch residues into soil after crop harvest might also promote residue accumulation because of nutrient limitations that restrict microbial activity, as observed for polybutylene adipate terephthalate (PBAT) in particular. The authors also noted that, to date, field-based studies have largely focused on the overall impacts of mulch residues on soil and crop health, and less attention has been given to their in-field degradability.

Biodegradable polymers derive their biodegradability from the presence of hydrolysable chemical bonds (e.g., ester, amide, and carbonate linkages) that are susceptible to microbial attacks through multiple biochemical pathways [8]. Under field conditions, the degradation of biodegradable mulch films typically proceeds through three main phases, whose rates and extent are strongly modulated by soil moisture, temperature, oxygen availability, pH, and soil microbial community. The initial phase, **biodeterioration**, involves physical/abiotic weathering and enzymatic activity, during which microbial colonization and biofilm formation induce early surface alterations, particularly under warm and moist conditions [2,6,8,10]. This is followed by **biofragmentation,** characterized by the progressive breakdown of polymers into smaller particles, i.e., depolymerization into lower molecular weight compounds (oligomers, dimers, and monomers) mediated by extracellular enzymes produced by bacteria and fungi. Under field conditions, the main chemical degradation process is hydrolysis; unlike the conventional films, the rate of hydrolysis depends mostly on the type of biodegradable polymer. In the final phase, these compounds are either **assimilated** into microbial biomass or **mineralized** through microbial respiration into CO_2_, H_2_O, and other inorganic products, with mineralization rates depending on soil temperature regimes, moisture dynamics, and the functional diversity of soil microbial consortia. For simplicity, the three phases of mulch decomposition are hereafter collectively referred to as (bio)degradation, and this term is used throughout the text.

Although laboratory-based studies using **direct methods** have been widely conducted to assess the (bio)degradation rates of mulch materials, comparatively few studies have evaluated these processes under field conditions, where degradation is typically assessed using **indirect methods**. According to Francioni et al. [2], methods used to estimate the in-field (bio)degradation of mulch materials include **visual analysis** (e.g., photographic analysis), **mass loss** measurements using mesh bags, **spectroscopic techniques** to detect changes in polymer structure following abiotic and biotic degradation, and the measurement of **CO_2_ release**. The development of standardized field test protocols is essential for reliably assessing the (bio)degradability of both conventional plastic and biodegradable mulches under diverse environmental conditions, and for predicting their (bio)degradation dynamics. Among these approaches, the mesh bag method provides a practical and widely applicable means of quantifying mass loss under field conditions. Spectroscopic techniques, in turn, offer valuable complementary information by enabling the detection of chemical and structural changes in the polymers [10,11], thereby improving the assessment of the degradation status of the original materials.

The main objective of this study was to assess the in-field degradation rates and predict biodegradation dynamics of two biodegradable mulch materials, a Kraft^®^ paper mulch and a flexible black Kritifil^®^ PBAT-based film (14 µm thick), compared with two conventional plastic materials, a black PP matrix geotextile fabric and a black PE film (40 µm thick), over approximately one year (361 days) under Mediterranean field conditions. The fate of mulch films will be assessed using physical (%mass loss) and chemical (ATR-FTIR spectroscopy) approaches, and the influence of edaphoclimatic parameters will be evaluated.

We hypothesized that the biodegradable mulch materials placed in nylon mesh bags, particularly the Kraft^®^ paper and Kritifil^®^ PBAT-based mulch, would degrade more rapidly and exhibit a substantially greater %mass loss over the experimental period than the conventional plastic materials (PP and PE). Although polyester materials, such as PBAT and cellulose-based (paper) materials, are considered biodegradable, their biodegradation rates are strongly influenced by factors including material morphology (e.g., crystallinity), chemical characteristics (e.g., molecular weight, bond stability, and additives), film thickness, the presence and activity of soil microorganisms capable of decomposing these materials, and favourable environmental conditions [2]. In addition, abiotic degradation processes (temperature, moisture) may represent a critical preliminary step in the overall degradation of these materials.

## 2. Results and Discussion

### 2.1. Monitoring of Soil Properties

**Figure 1:**
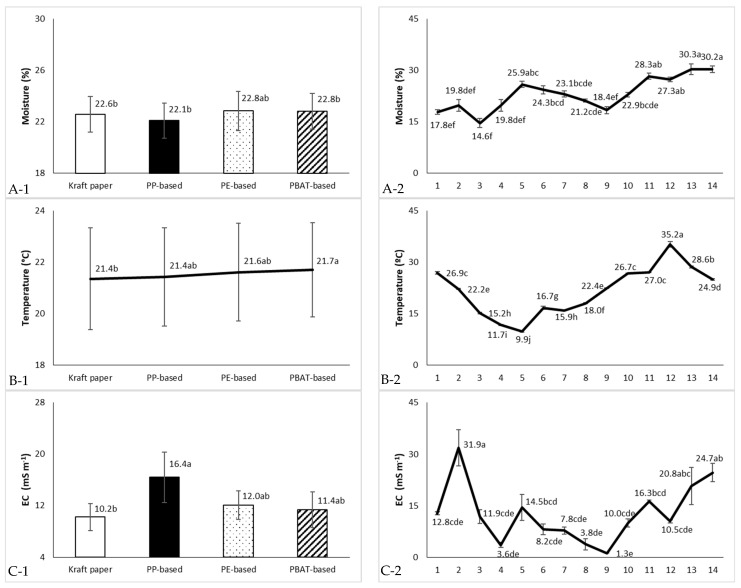
Monthly variation in moisture content (%) (**A**), temperature (°C) (**B**) and electrical conductivity (CE, mS m^−1^) (**C**) in soil, at 0–20 cm depth, beneath the different materials (**A-1**, **B-1**, **C-1**) and throughout the experimental period (**A-2**, **B-2**, **C-2**) (treatments: 1 = Kraft^®^ paper, 2 = PP-based geotextile fabric, 3 = PE-based mulch, 4 = Kritifil^®^ PBAT-based mulch; monthly dates: 1 = September 2024 to date 14 = October 2025). Vertical bars denote standard error (SE).

By measuring soil edaphoclimatic parameters directly beneath the mulch layers, the effects of mulch materials were effectively isolated from external climatic factors such as UV radiation, wind, and air temperature. Figure 1A–C present the soil monitoring data, including moisture (%), temperature (°C), and electrical conductivity (EC, mS m^−1^), which represented the main edaphoclimatic variables potentially influencing mulch degradation, independent of the external climate.

**Figure 2:**
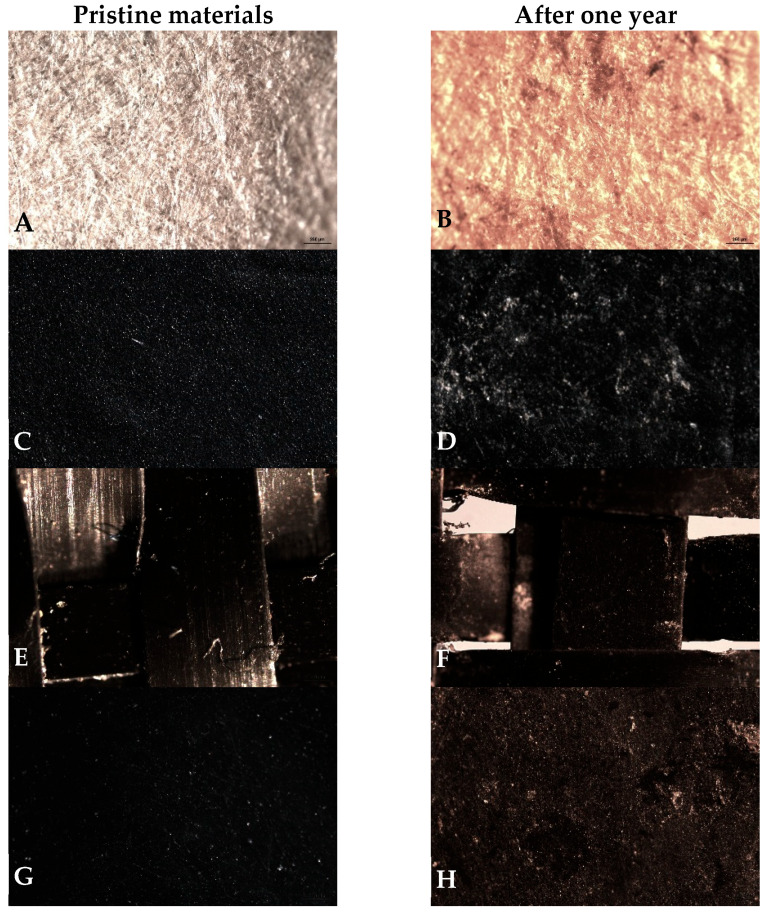
Structural characteristics of mulch materials before and after field exposure. Pristine materials are shown in (**A**,**C**,**E**,**G**), and materials after one year of field exposure in (**B**,**D**,**F**,**H**). Images were obtained at a scale of 250 µm using a Leica DM6 B microscope equipped with a K3C colour camera (Leica Microsystems, Germany). The materials included Kraft^®^ paper (**A**,**B**), Kritifil^®^ PBAT-based biofilm (14 µm thickness) (**C**,**D**), PP-based geotextile fabric (**E**,**F**), and PE-based film (40 µm thickness) (**G**,**H**).

Regarding the variation in soil moisture during the seasonal period under analysis, mulch materials maintained overall similar soil moisture levels, averaging 22.6% (Figure 1(A-2)). Soil temperature decreased steadily from 27.3 °C in September 2024 to 9.7 °C in January 2025 (Figure 1(B-2)), then increased significantly by August 2025. The mean annual soil temperature was the highest under the black PBAT biofilm (21.7 °C), whereas the lowest temperature was recorded beneath Kraft^®^ paper (21.4 °C) (Figure 1(B-1)), in agreement with [3,12]. Overall, Zhang et al. [3] also found increased soil temperature and moisture under the PE mulch, in comparison with bare soil, and Machado et al. [13] reported improved soil temperature regulation with green geotextile fabric mulch, comparable to pine bark, in the cultivation of blueberry ‘Ozarkblue’ and ‘Legacy’ under the warm conditions in southern Portugal, relative to bare soil and black or white geotextile fabrics.

Although point-in-time temperature measurements may underestimate daily maxima, the standardized protocol allowed for consistent and unbiased comparisons among mulch treatments. Based on monthly temperature measurements, the estimated cumulative thermal time (°day, T_base_ = 0 °C) [8,14,15] over the study period followed the order:

Kritifil^®^ PBAT: 9245.43 > PE: 9208.50 > PP: 9131.83 > Kraft^®^ paper: 9106.83°day.

Black mulches (PBAT and PE) promoted greater soil warming, resulting in higher mean annual temperature (21.6–21.7 °C, respectively) and greater estimated cumulative thermal time (PBAT: 9245.43 and PE: 9208.50°day), compared with PP and Kraft^®^ paper (9131.83 and 9106.83°day, respectively). These results confirmed the findings by Tofanelli et al. [5] and Sintim et al. [12]. The slightly lower soil temperatures observed under Kraft^®^ paper in the present study are likely attributable to its lighter colour and higher reflectance, which reduced heat absorption and led to the lowest annual thermal accumulation [5,16], and may help to prevent root-zone stress in summer. In contrast, the PBAT biofilm consistently maintained marginally higher soil temperature than PE, resulting in the greatest estimated cumulative thermal exposure over the study period. These results indicated that the relevance of thermal time for mulch degradation is material-dependent. While it has limited explanatory power for cellulose- and PBAT-based mulches, whose degradation is primarily governed by moisture-driven microbial activity, it remains an important factor for plastic mulches, where temperature-dependent processes such as thermo-oxidative and photo-induced degradation occur.

Among the various treatments, the black PP-based geotextile was distinguished with an average EC of 16.4 mS m^−1^ (Figure 1(C-1)) and a very high salt concentration in October 2024 (33.0 mS m^−1^) (Figure 1(C-2)), possibly due to a greater salt accumulation on the soil surface layer, associated with a lower soil moisture resulting from the porous structure of the material. In November 2024, following the cessation of fertilization but with continued irrigation and rainfall, EC decreased to a value similar to that of the other treatments (17.7 mS m^−1^) (no significant interaction between the treatments and the date was observed).

### 2.2. In Situ Degradation of Conventional Plastic and Biodegradable Mulch Materials: Mass Loss

Field-based studies evaluating the degradation and biodegradation rates of mulch materials have gained increasing importance in recent years, following the FAO’s [17] call for the urgent replacement of conventional plastics with biodegradable alternatives [6,7,18]. The (bio)degradation rates of the four mulch films were assessed by measuring mass loss (%), an indirect indicator of degradation [6]. An overestimation of mass loss might have occurred if changes in film thickness during the (bio)degradation process allowed pieces to escape through the mesh bag holes into the soil. To minimize these losses, care was taken during laboratory washing using a small sieve (0.5 mm) to recover minor pieces [2]. Table 1 shows that not all intact pieces were recovered from the mesh bags after approximately 12 months in the field in the surface soil. The lowest number of recovered pieces was observed for paper (approximately 51% recovered), followed by the geotextile fabric (approximately 67% recovered). Some of the recovered material exhibited clear physical (visual and stereo microscope) signs of degradation, particularly in the paper and, to a lesser extent, the PBAT-based material and geotextile fabric (Figure 2).

Kraft^®^ paper showed an apparently high degree of degradation, with mass loss reaching 72 ± 18.5% within one year, whereas PBAT showed a significantly minimal mass loss (3.5 ± 5.7%) over the same period, despite being exposed to the highest estimated cumulative thermal time (9245.43°day) and relatively stable soil moisture. Consistent with the present findings, Sintim et al. [1] reported a nearly complete degradation of paper (approximately 100%) in mesh bags not placed beneath soil covers within six to twelve months across both warmer and cooler regions, whereas PBAT exhibited low to moderate biodegradation rates (26 to 83%) over 36 months under the same conditions. Zhang et al. [3] reported that evidence of PBAT and PLA mulch material (bio)degradation was very slow on topsoil, showing little to no (bio)degradation within the first four months. In addition, Coutris et al. [8] reported mass losses for PBAT-based mulch materials in mesh bags varying from 8% to 48% after 12 months, respectively, in a cold Norwegian climate, with little difference in mass loss at both the 10 and 30 cm soil depths. Applying the zero-order kinetics model (negative quadratic model), these authors estimated that 90% mass loss would take 21–58 months, demonstrating the potential biodegradability of PBAT-based mulch, as defined in EN norm [4].

PBAT exhibited the lowest “mass loss” among biodegradable mulches while polyolefins (PP and PE) showed negligible degradation over the study period. The PBAT-based mulch was highly associated with the Mean Enzymatic Index (MEI) and basal respiration rate in the surface soil beneath the mulch. This suggested an overall stimulation of soil microbial activity and a relatively balanced contribution of key soil enzymes, namely dehydrogenase, β-glucosidase, alkaline phosphatase, and arylsulfatase. Senevirathne et al. [10] also found an increased β-glucosidase activity under the PBAT film in field conditions. However, enzymes directly involved in PBAT biodegradation, such as esterase, especially the cutinase, and lipase, were not measured in the present study; therefore, the effective capacity of soil to enzymatically degrade this copolymer could not be directly inferred from the present data. Moreover, the relatively low di(2-ethylhexyl) phthalate (DEHP) concentration in the PBAT (2.64 ± 0.52 mg kg^−1^; Table 2) would not be expected to reduce polymer breakdown under field conditions [19].

In contrast, PP and PE mulch pieces showed no evidence of degradation after one year in the field. The negative “mass loss”, i.e., mass gain values observed (Table 1), likely reflected potential microbial colonization and/or adhesion of soil particles to the mulch surface, resulting in apparent increases in recovered mass rather than true mass loss (bio)degradation. Similar mass gain results were observed by Sintim et al. [1] for PE-based mulch pieces in mesh bags across two different field conditions, and by Graf et al. [9] for PBAT-based materials.

### 2.3. Evidence of Deterioration Based on ATR-FTIR Spectral Changes

**Figure 3:**
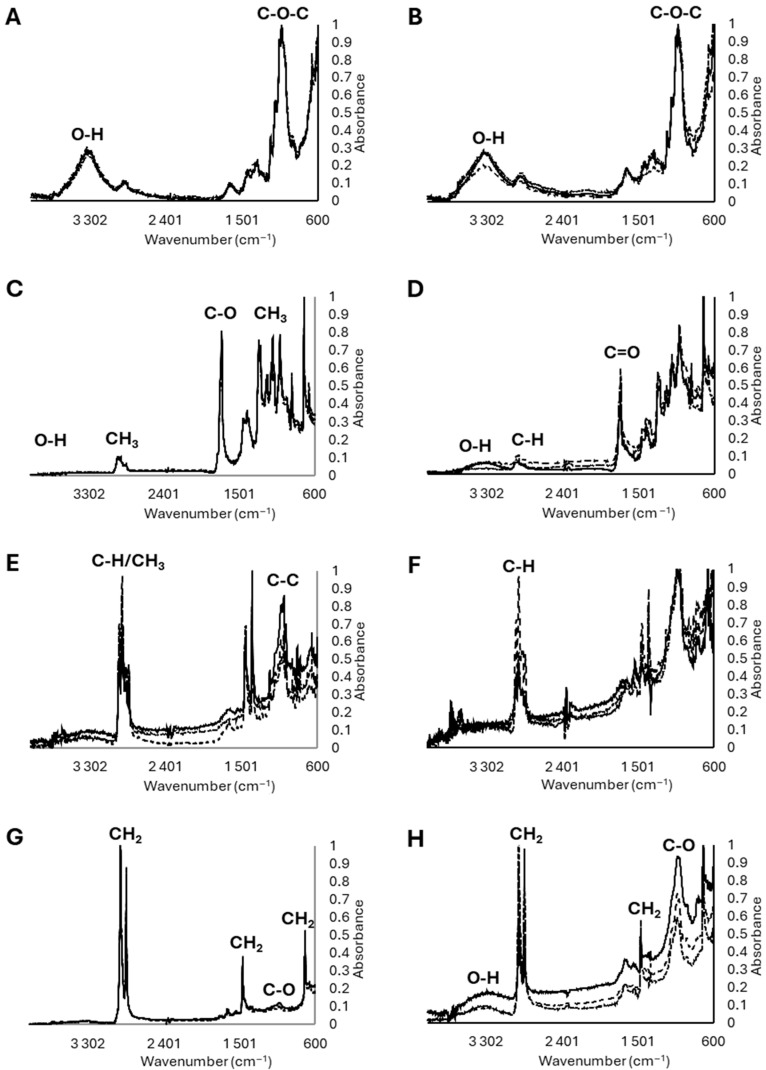
FTIR spectra of mulch polymers: pristine materials at the start of the experiment (**A**,**C**,**E**,**G**), with major functional groups indicated, and corresponding spectra after one year of in situ field exposure (**B**,**D**,**F**,**H**). The spectra illustrate the relative degradation of the different materials: Kraft^®^ paper (**A**,**B**), Kritifil^®^ PBAT-based biofilm (14 µm thick-ness) (**C**,**D**), PP-based geotextile fabric (**E**,**F**), and PE-based film (40 µm thickness) (**G**,**H**). Replicates are presented in different dashed lines.

Mediterranean climatic factors, such as wind and UV radiation, did not directly influence mulch pieces degradation on this study because the nylon mesh bags containing the mulch pieces were placed beneath the respective soil covers, protecting them from direct exposure. Under these conditions, the (bio)degradation of mulch pieces in nylon mesh bags, including both conventional plastic and biodegradable films, was assumed to be primarily influenced by their chemical composition and soil quality. In fact, some mulch pieces (1.5 × 1.5 cm each) retrieved from the mesh bags after one year in the field exhibited clear visible signs of deterioration (Figure 3B,D,F,H), particularly the Kraft^®^ paper (cellulose) and PP-based geotextile mulch.

The ATR-FTIR analysis confirmed that pristine Kraft^®^ paper samples (Figure 3A) consisted primarily of cellulose fibres [20]. The spectra showed a broad O–H stretching band at approximately 3600–3000 cm^−1^, C–H stretching bands at approximately 3000–2800 cm^−1^, a band at approximately 1649 cm^−1^ commonly associated with H–O–H bending of absorbed water in cellulosic materials, characteristic cellulose bands at approximately 1435 cm^−1^ (CH_2_ scissoring), approximately 1370 cm^−1^ (C–H bending), approximately 1035 cm^−1^ (C–O/C–O–C stretching in the polysaccharide ring), and approximately 903 cm^−1^ (β-glycosidic linkages between glucose units). All spectra were normalized to the area of the band at 1024 cm^−1^**,** ensuring comparability across replicates.

After one year of field exposure, the main cellulose bands identified in pristine samples (Figure 3A), prior to field exposure, were largely preserved in the recovered material (Figure 3B). However, an increase in the intensity of the O–H stretching band centred approximately at 3400 cm^−1^ was observed, suggesting an increased hydroxyl content and/or higher moisture adsorption (cellulose is hygroscopic). As noticed, the Kraft^®^ paper biofilm (cellulosic material) exhibited the highest mass loss (72 ± 18.5%) after one year in the field (Table 1 and Table 3), consistent with its hydrophilic nature and a high susceptibility to microbial colonization and hydrolytic degradation. This process was primarily associated with the cleavage of glycoside bonds (Figure 3B; Table 3) and was promoted by moisture availability and soil enzymatic activity, despite the slightly lower cumulative thermal exposure compared with PBAT (9106.83 vs. 9245.43 °day; Δ = 138.6°day). These results indicated that polymer composition and the presence of functional groups susceptible to microbial attack were the main drivers of (bio)degradation under field conditions, considering that soil temperature (21.4 °C) and moisture level (22.6%) were generally favourable for microbial activity.

For the pristine Kritifil^®^ PBAT-based material (Figure 3C), characteristic FTIR bands were observed at 2950, 2915, and 2838 cm^−1^ (methylene C–H stretching), at 1709 cm^−1^ (ester carbonyl, C-O stretching), 1504 and 1456 cm^−1^ (aromatic ring vibrations), and at 1243 and 1099 cm^−1^ (C–O–C stretching of ester groups). Bands in the 700–900 cm^−1^ region, including approximately 725 cm^−1^, were consistent with out-of-plane aromatic C–H, associated with terephthalate units. These assignments confirm the typical PBAT polymer backbone structure [8,21,22].

After one-year of soil exposure (Figure 3D), the ATR-FTIR spectra of PBAT-based mulch pieces indicated a slight decrease in the ester carbonyl (C=O) band together with a reduction in the ester C–O stretching region, accompanied by an increase in the O–H stretching band (approximately 3200–3600 cm^−1^). This finding is consistent with the great decrease in the C-O peak area after belowground exposure compared to aboveground exposure due to the enzymatic hydrolysis of ester bonds observed by Graf et al. [9]. As PBAT is a polyester containing hydrolysable ester linkages, these spectral changes are consistent with early-stage hydrolysis, which cleaves ester bonds, shortens polymer chains and produces hydroxyl and carboxylic acid end groups. The relatively small magnitude of these changes is indicative of degradation remaining limited to superficial hydrolysis, occurring primarily on the surface of the polymer rather than in the form of extensive bulk degradation. Together, the spectral modifications indicate limited depolymerisation through hydrolytic processes under soil moisture conditions, without evidence of extensive bulk polymer degradation [8]. Previous studies have similarly reported hydrolysis as a dominant mechanism in PBAT degradation, together with natural weathering, phytochemical transformation, mechanical wear, and microbial activity under open-field conditions [10]. On the other hand, the relative stability of PBAT in the present study was primarily attributable to its low hydrophilicity, reduced accessibility of the polymer matrix to water and microorganisms, and the requirement for specific soil microbial enzyme esterases for efficient depolymerisation, rather than to polymer thickness (14 µm) or the relatively low concentration of low additive (2.64 ± 0.52 kg kg^−1^) [19].

Overall, Kraft^®^ paper showed pronounced mass loss, indicating active hydrolytic and microbial processes, while, in PBAT, the O–H band primarily indicated limited water absorption, with no clear evidence of chemical or enzymatic degradation, consistent with its relative structural stability under the conditions tested. In contrast to Kraft^®^ paper, PBAT exhibited significantly lower mass loss (3.5 ± 5.7%) after one year of field exposure (Table 1 and Table 3). Hydrolysis reduced mass when cleaved polymer fragments detached from the polymer surface. The extent of mass loss depended on the number of polymer chains cleaved and the depth to which hydrolysis penetrates.

The ATR-FTIR spectra of pristine geotextile fabric (Figure 3E) consisted primarily of a non-polar polyolefin (PP), with no other polymers unambiguously detected. The characteristic bands were observed at ≈2950 cm^−1^, 2915 cm^−1^, and ≈2838 cm^−1^ (CH_3_/CH_2_ stretching), ≈1449 cm^−1^ and 1375 cm^−1^ (C-H bending and CH_3_ symmetric deformation), ≈1166 cm^−1^ (C-H bending/in-plane CH_3_ deformation, and C–C stretching). Additional bands at ≈997 cm^−1^ and 972 cm^−1^ were assigned to in-plane CH_3_ deformation and C–C stretching, while bands at ≈841 cm^−1^ and 808 cm^−1^ corresponded to in-plane C-H/CH_2_ deformation and C–C/C-C-H stretching. Jung et al. [23] also observed similar spectra for PP-based materials.

ATR-FTIR analysis of the recovered PP-based samples after exposure (Figure 3F) showed pronounced oxidation-related surface changes, while the main PP fingerprint bands remained identifiable, suggesting that ageing was largely confined to the near-surface region rather than reflecting extensive bulk polymer breakdown. In fact, exposed PP-based pieces exhibited early surface ageing, evidenced by the appearance of oxygenated features, including a broad O–H band between ≈3300 and 3700 cm^−1^, consistent with limited surface oxidation and/or moisture adsorption. Changes were also observed in the carbonyl region (≈1710–1740 cm^−1^), consistent with early oxidative abiotic degradation process of the surface material. A slight reduction in the relative intensity of the C–H stretching bands at ≈2950, 2915, and 2838 cm^−1^ was detected, which may reflect surface chemical modification rather than extensive bulk polymer degradation. These features are consistent with early-stage abiotic weathering (surface oxidation), rather than true biodegradation [24,25,26]. However, similar alterations may also arise from the presence of biofilms; therefore, they cannot be unequivocally attributed to polymer oxidation alone. [27].

Despite these minor surface-level spectral variations, PP-based material exhibited essentially no net mass change (−1.7 ± 17.4%), though the great variability among replicates under the field conditions, indicating that overall, the bulk polymer remained intact. This suggested that the observed ATR-FTIR changes predominantly reflected surface oxidation and/or surface fouling (biofilm/particle adhesion), and the bulk polymer remained chemically and physically intact in bulk, without a corresponding decrease in bulk polymer mass. ATR-FTIR analysis of the recovered PP samples (Figure 3F) showed pronounced oxidation-related surface changes, while the main PP fingerprint bands remained identifiable. This suggests that ageing was largely confined to the near-surface region rather than reflecting extensive bulk polymer breakdown. Nevertheless, these changes may also be associated with microbial colonization at the polymer surface, making it difficult to distinguish between true polymer oxidation and biofilm-related signals on PP and PE surfaces [27]. Furthermore, the relatively high DEHP concentration measured in the geotextile fabric (32.3 ± 7.6 mg kg^−1^; Table 1) may have influenced weathering and/or biological interactions under field conditions [19].

Unlike Kraft^®^ paper and PBAT, PP-based mulch pieces exposed for one year under the same field conditions were not expected to undergo hydrolytic chain scission because PP lacks hydrolysable functional groups.

Polyethylene is also a non-polar polyolefin composed of repeating –CH_2_–CH_2_– units and consists almost exclusively of C–C and C–H bonds (Figure 3G). The ATR-FTIR spectra of the pristine samples confirmed the presence of PE-based fibres [28]. The main absorption bands were observed at 2916 cm^−1^ and 2847 cm^−1^ assigned to C–H stretching vibrations, and smaller bands at 1472 cm^−1^ and 1462 cm^−1^ assigned to CH_2_ bending vibrations. Additional bands at 729–717 cm^−1^ were observed, characteristic of CH_2_ rocking/in-plane deformation typical of PE.

After field exposure (Figure 3H), FTIR analysis showed that the main characteristic bands of PE-based were largely preserved, indicating that the bulk polymer structure remained intact. However, a broad band at 3200–3600 cm^−1^, attributable to O-H stretching (e.g., hydroxyl, hydroperoxide groups formation, and/or absorbed water) appeared. This feature suggested the occurrence of a possible initial (limited) surface oxidation of PE-based mulch and/or adsorbed water from soil, suggesting oxidative ageing. A high increase in absorption in the carbonyl region was also observed (approximately 1710–1740 cm^−1^), which may indicate the initial formation of carbonyl (C=O) groups, associated with incipient oxidative processes. These reactive oxygen species (ROS) could react with surface CH_2_ groups, leading to the formation of oxygen-containing functional groups. Such processes were expected to be restricted to the polymer surface and do not imply bulk material degradation. Additionally, the adsorption of soil organic matter may contribute to the observed C–O spectral features.

The negative “mass loss”, i.e., net mass gain measured for PE-based pieces (–11.3 ± 6.6%, Table 1 and Table 3), likely reflected adsorption of soil particle/biofilm and retained moisture. The ATR-FTIR evidence of oxygenated groups supported surface oxidation, which apparently increased surface wettability and promoted adsorption/colonization, indirectly contributing to the apparent mass gain. The formation of hydroxyl and C–O groups on the surface increases polarity, which makes soil particles, organic matter, and microorganisms adsorb more easily at mulch surface, resulting in a measurable mass increase.

ATR-FTIR and mass measurements suggested that both PE- and PP-based experienced early surface ageing under soil exposure, but the effects were slightly more pronounced for PE-based material, i.e., slightly more polar, with more adhesion of soil particles and biofilms and mass gain potential than the PP-based material, consistent with greater surface oxidation and enhanced adhesion on PE. Overall, these observations reflected distinct degradation pathways driven primarily by chemical structure pathways, rather than being strongly affected by soil temperature or moisture. Kraft^®^ paper (cellulose) and PBAT could interact with water through hydroxyl and ester functionalities, enabling moisture-dependent processes such as swelling and hydrolysis (particularly for PBAT) (Table 3). In contrast, PP- and PE-based lack hydrolysable functional groups and therefore degraded predominantly via oxidative mechanisms (Table 3). Taken together, the mass-change results and ATR-FTIR analyses highlighted contrasting behaviours among cellulose, PBAT-, PP-, and PE-based materials under the tested field conditions [26,29,30,31]. Although high activities of soil dehydrogenase, β-glucosidase, alkaline phosphatase, and arylsulfatase confirmed that the experimental soil was biologically active, these general soil enzymes were not directly involved in polyester (PBAT) depolymerization. PBAT degradation typically which relies on specialized microbial esterase, particularly the cutinase- and lipase-like enzymes [32], which were not assessed in the present study. This highlights a potential gap between laboratory biodegradability certification and in-field degradation performance.

## 3. Materials and Methods

### 3.1. Study Design

This study was conducted at the Innovation Pole of Fataca in Odemira, southern Portugal (37.5903° N, 8.7403° W). It was conducted in a Mediterranean climate with dry summers and wet winters. Over the past 30 years, the region has experienced mean annual air temperatures of approximately 19 °C, with minimum and maximum values ranging from 7 °C to 31 °C. Mean monthly rainfall varied from about 3 mm in July and August to 75 mm in December, with an average annual rainfall of 437 mm. These climatic conditions were considered favourable for blueberry cultivation.

**Figure 4:**
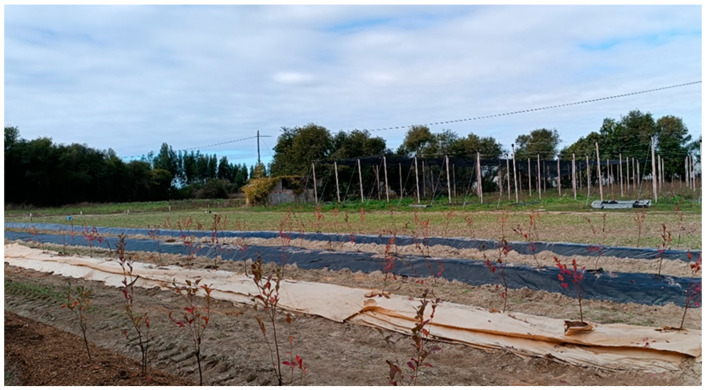
Partial view of the experimental site with blueberry ‘Centra Blue’ planted in raised beds covered with different mulch types, at Fataca, in southern Portugal: (1) paper Kraft^®^; (2) black PP-based geotextile fabric; (3) black polyethylene, PE-based, 40 µm thickness; (4) black PBAT-based Kritifil^®^, 14 µm thickness.

In June 2023, two-year-old blueberry plantlets (*Vaccinium virgatum Aiton* cv. Centra Blue), a deciduous rabbiteye cultivar developed recently (*V. ashei* J.M Reade syn. = *V. virgatum* Aiton) by the Horticulture and Food Research Institute of New Zealand Limited (HortResearch) [33], were manually transplanted from a nursery to the field (Figure 4). The experimental layout consisted of four mulch treatments manually installed on the surface of raised beds cultivated with ‘Centra Blue’ blueberry plants. The treatments were arranged in a randomized complete block design with three replicates; each replicate was composed of five plants. Raised beds measured 0.30 m in height, 1.60 m in width, and 15 m in length. Each planting row was equipped with two fertigation lines fitted with emitters delivering 2.0 L h^−1^ at 30 cm spacing. Fertigation was applied according to crop requirements, two to three times a day for 10 min each, from March to November, supplying ammonium sulphate, potassium sulphate, magnesium sulphate, and monoammonium phosphate. The EC of the nutrient solution was maintained below 12 mS m^−1^ to avoid salinization.

The mulch treatments were: (1) Kraft^®^ paper biofilm, (2) commercial black PP-based geotextile fabric, (3) commercial black PE-based plastic film (40 µm thickness), and (4) black Kritifil^®^ PBAT-based biofilm (14 µm thickness). The Kraft^®^ paper biofilm was a cellulose-based biofilm supplied by COTESI^®^ (Companhia de Têxteis Sintéticos S.A., Alijó, Portugal). The geotextile fabric is composed of woven PP-based fibres (woven 50 × 70.97 g m^−2^; tensile strength 85 ± 0.05 kgf/5 cm; [34]) and has been widely used in blueberry plantations across the Mediterranean region. It is relatively dense yet permeable to water and air (12 ± 2 L m^−2^ s^−1^; [35]), while effectively blocking light to suppress weed growth. The black PE-based film, the most commonly used plastic mulch in agriculture, was applied with a thickness of 40 µm to ensure durability for at least two years in the perennial crop plantation. The Kritifil^®^ film was a black PBAT-based copolymer, composed of adipic acid, 1,4-butanediol, and terephthalic acid, with a thickness of 14 µm (no thicker material was available), and represents a potential biodegradable alternative film.

Mulch materials, both synthetic and those of organic origin, often contain additives in their formulation, particularly to enhance UV resistance, as well as to provide plasticizing effects, coloration, texture, and other functional properties [19]. The phthalate plasticizer, namely the DEHP, is used to make plastics softer, flexible, and durable. In the present study, the DEHP concentration in mulch materials (Table 2) was extracted from the mulches by liquid extraction with organic solvents (n-hexane and dichloromethane) and determined by gas chromatography analysis. Due to phthalate ability to migrate into soil and crops, they may contribute to environmental contamination and potential human exposure associated with endocrine and reproductive health effects [36].

### 3.2. Monitoring Soil Moisture, Temperature, and Salinity

**Figure 5:**
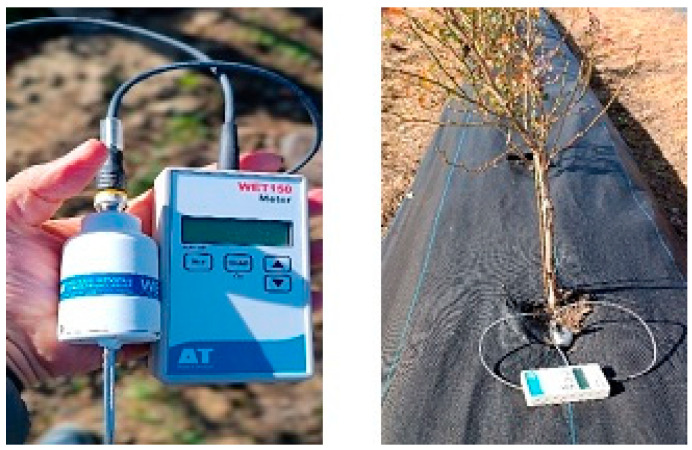
Aspects of soil sensor Delta-T^®^ WET150, Soil Moisture KIT measurements in the field.

The effects of different mulch types on edaphoclimatic conditions were monitored taking a single-time monthly measurement between 10.30 and 11.00 a.m., from 1 September 2024 to 14 October 2025. Measurements were taken at a depth of 0–20 cm beneath each mulch type, adjacent to irrigation tubing and in close proximity to plant roots, ensuring that the recorded microclimatic conditions reflected the environment immediately surrounding the mulches. The parameters included the soil moisture content (%), soil temperature (°C), and salinity (EC, mS m^−1^) and were assessed using a Delta-T^®^ WET150, Soil Moisture KIT (Delta-T Devices, Cambridge, UK) (Figure 5). These results were mostly discussed in [37]. Soil temperature data were used to characterize seasonal thermal conditions [14] under the different mulch treatments. Owing to the monthly sampling frequency, only the cumulative thermal time (°day) was estimated. Calculations used a base temperature (T_base_) of 0 °C, reflecting the threshold below which root growth and soil microbial and enzymatic activity are considered negligible. Growing degree-days (°day), also referred to as thermal time or heat units, were calculated by summing, over time, the difference between observed temperatures and the base temperature [14,15,38]. This approach provided a measure of heat accumulation during the experimental period and was used with averaged sampled temperature data to assess temperature-related processes.

### 3.3. Mass Loss: Mesh Bag Technique

Mulch (bio)degradation was evaluated using a combination of mass loss measurements and polymer structural analysis. The nylon mesh bag technique [1,15,39] was applied on the surface of raised beds at the Fataca farm. Each white mesh bag (20 × 20 cm) contained 30 pieces of plastic or biodegradable mulch films (1.5 × 1.5 cm each), and had openings of approximately 1 mm to allow water, gas, and microbial access for mulch biodegradation, while retaining the material and maintaining bag integrity throughout the 12-month field experiment [6,40]. In previous studies, Sintim et al. [1] used mesh bags with 250 µm openings to minimize material loss while still allowing for microbial degradation, while Graf et al. [9] tested nylon mesh bags with 100 µm and 5 mm openings to evaluate microbial decomposition, and meso- to macrofauna-mediated physical degradation; however, they did not observe physical degradation after 12 months under temperate field conditions.

**Figure 6:**
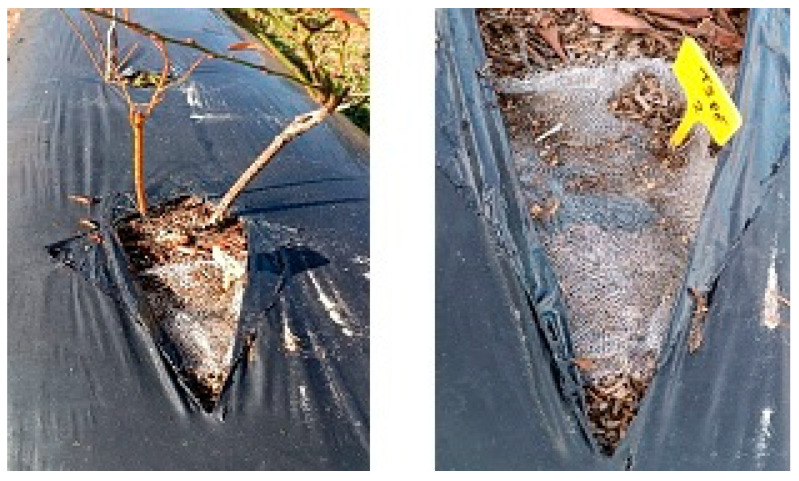
Aspects of nylon mesh bags containing mulch pieces, placed at surface soil of raised beds at Fataca farm, in Odemira (south Portugal).

In October 2024, the nylon mesh bags were placed horizontally, in triplicate, on the surface of raised beds, beneath the corresponding mulch material, close to the plants and irrigation drippers, and fixed to the soil (Figure 6). The mesh bags remained in the field for about one year (361 days). In October 2025, mesh bags were carefully recovered from the field, transported to the laboratory, gentle and carefully washed with distilled water [6], using a small sieve to recover fragments and avoid both underestimation (by leaving too many soil particles adsorbed) or overestimation of mass loss (by destroying or losing the material) [2,9,10,40], and washed pieces were counted, air-dried to constant weight, and weighed with an precision balance, as also reported by Bianchini et al. [6].

In each nylon mesh bag, mass loss of mulch pieces was determined and expressed as the percentage (%) of initial weight and calculated according to (1) [1,6]:

{[initial weight (mg/bag) − final weight (mg/bag)]/initial weight (mg/bag)} × 100(1)

Salomez et al. [32] considered the mass loss method the most relevant and accurate to assess the degradation of mulch material.

### 3.4. ATR-FTIR Analysis of Polymers Degradation Following Field Exposure

To assess potential polymer degradation in field, recovered mulch fragments were analyzed with ATR-FTIR (Attenuated Total Reflectance–Fourier Transform Infrared) spectroscopy. The spectra of the recovered materials were compared with those of the corresponding pristine (initial) polymers to identify alterations indicative of degradation and to evaluate the extent of polymer breakdown under field conditions. FTIR analysis (FTIR IR Affinity, Shimadzu Corporation, Kyoto, Japan) with an ATR accessory provided information on chemical and structural changes in polymer matrices, including bond breakage, observed as a decrease in peak intensity or disappearance of specific peaks in the infrared spectrum, often indicating that a particular chemical bond has been broken, as well as the potential formation of new functional groups.

Polymer spectra were recorded over 600–4000 cm^−1^**,** with 64 scans per sample at a 4 cm^−1^ resolution, as also performed by [10]. Background spectra were collected for each mesh sample, and subtracted atmospheric correction, and normalization in IR Solution software (version 1.60, Shimadzu Corporation, Kyoto, Japan). ATR-FTIR spectra of recovered mulch pieces were compared with the control (initial mulch) spectra to identify peaks that had either disappeared, emerged or exhibited alterations in their intensity. For each polymer, three sub-samples were analyzed to ensure reproducibility, and the mean spectral values were used to visualize the compositional changes for each type of mesh mulch material.

### 3.5. Statistical Analysis

All data were presented as means ± standard deviation (n = 3) for each treatment: Kraft^®^ paper biofilm, PP-based geotextile fabric, black PE based film, 40 µm thickness, and PBAT matrix Kritifil^®^ biofilm (14 µm thickness).

Prior to statistical analysis, all variables were tested for normality and homogeneity of variances. Normality was assessed using the Shapiro–Wilk test in the Descriptive Statistics: Test for Normality module of Statistica^®^ version 12 [41]. The homogeneity of variances was evaluated using Levene’s test, included in the one-way ANOVA procedure. For all variables, the Shapiro–Wilk test indicated that the data did not significantly deviate from normality (*p* ≥ 0.05), and Levene’s test confirmed homogeneity of variances (*p* ≥ 0.05). Consequently, one-way ANOVA was applied to assess differences among treatments, followed by post hoc comparisons when appropriate, using the robust Bonferroni test for significant effects (*p* < 0.05).

## 4. Conclusions

The present findings confirmed the hypothesis that biodegradable mulches degraded faster than conventional plastic films under present field conditions. Among the tested materials, Kraft^®^ paper degraded more rapidly after one year, with a controlled mean temperature of 21 °C and moisture content of 22%, followed by PBAT-based film, whereas PP-based geotextile fabric and PE-based film remained largely intact. Polymer composition and inherent biodegradability of paper and PBAT-based films, primarily mediated by microbial processes, were the main drivers of in-field degradation, whereas cumulative soil temperature and other microclimatic factors played only a minor role. The present results highlighted the persistence of non-biodegradable plastics and emphasized that intrinsic polymer chemistry and accessibility to enzymatic activity largely determined degradation rates. Further studies on soil microbial diversity and interactions with mulch types would contribute for a more comprehensive understanding of the mechanisms involved in the degradation process.

From a practical perspective, this study provided guidance for the selection and management of sustainable mulch materials in irrigated perennial cropping systems under Mediterranean conditions and demonstrated that the combined use of field-based mass loss measurements (mesh bag technique) and ATR-FTIR spectroscopy offers a robust approach for evaluating in-field degradation. This approach complements more labour-intensive techniques, such as microbial assays or CO_2_ evolution tests, and provided valuable insights into the physical, chemical, and biological processes underlying mulch degradation.

Present findings were specific to irrigated Mediterranean conditions and should therefore be extrapolated to other environments with caution. Future research should incorporate more frequent sampling and larger number of replicates, longer-term monitoring, and evaluation across a wider range of environmental conditions to better capture temporal dynamics and the influence of edaphoclimatic variability on mulch degradation.

## Figures and Tables

**Table 1:** Means ± standard deviation (n = 3) of the number of pieces, percentage mass loss and recovered mass (%) of plastic and biodegradable mulch pieces after 1-year of in situ decomposition on the soil surface.

Mulch Type	Number of Initial Pieces (1.5 × 1.5 cm)	Final Number of Pieces	Initial Mass (mg)	Final Mass (mg)	Mass Loss (%)	Recovered Mass (%)
Kraft^®^ paper	30	15.3 ± 6.1	1140 ± 261.5	305.57 ± 175.8	72.09 ± 18.5 ^a^	27.91 ± 18.5 ^b^
PP-based geotextile,	30	20 ± 4.6	920 ± 80	935.7 ± 78.6	−1.71 ± 17.4 ^b^	102.17 ± 7.4 ^a^
PE-based, 40 µm	30	28 ± 0	290 ± 30.6	325.07 ± 17.9	−11.25 ± 6.6 ^b^	111.25 ± 6.6 ^a^
PBAT-based Kritifil^®^, 14 µm	30	28.7 ± 1.5	240 ± 32.2	234.03 ± 23.9	3.51 ± 5.7 ^b^	96.49 ± 5.7 ^a^

PP = polypropylene; PE = polyethylene; PBAT = polybutylene-adipate-terephthalate; in the mass loss and recovered mass columns, different letters indicated significant differences according to Bonferroni test (*p* < 0.05).

**Table 2:** Mean ± SD concentration of DEHP phthalate in mulch materials.

Material	DEHP (mg kg^−1^ Dry Weight)
1- Kraft^®^ paper	0.02 ± 0.002
2- PP-based geotextile	32.30 ± 7.64
3- PE-based, 40 µm thickness	7.86 ± 0.82
4- PBAT-based Kritifil^®^, 14 µm thickness	2.64 ± 0.52

SD = standard deviation; DEHP = di(2-ethylhexyl) phthalate; PP = polypropylene; PE = polyethylene; PBAT = polybutylene adipate terephthalate.

**Table 3:** Summary table: mean values of mass loss (%), estimated cumulative thermal time (°day, T_base_ = 0 °C), soil moisture (%) and phthalate additive (mg DEHP kg^−1^), the degradation mechanism, and ATR-FTIR observation in different mulch materials.

Material	Mass Loss (%)	Estimated Cumulative Thermal Time (°day, T_b_ = 0 °C)	Soil Moisture (%)	DEHP (mg kg^−1^)	Proposed Degradation Process	ATR-FTIR Observation
Kraft^®^ paper	72	9106.83	22.6	0.02	Chemical (hydrolytic) and biodegradation	Functional groups (cellulose bands) preserved; increased O–H vibration (3400 cm^−1^)
PP-based geotextile	−1.71	9131.83	22.1	32.2	Surface-level chemical alteration (limited oxidation)	Functional groups largely unchanged
PE-based, 40 µm	−11.3	9208.50	22.8	7.86	Minor surface oxidative/ chemically stable	Functional groups preserved; high increased signal at 1117–953 cm^−1^
PBAT-based Kritifil^®^, 14 µm	3.5	9245.43	22.8	2.64	Minimal hydrolysis/chemically stable (possible enzyme limited biodegradation)	Slight O–H vibration increase (3400 cm^−1^); minor ester band changes

DEHP = di(2-ethylhexyl) phthalate; ATR-FTIR = Fourier Transform Infrared Attenuated Total Reflectance; PP = polypropylene; PE = polyethylene; PBAT = polybutylene adipate terephthalate.

## Data Availability

This study did not involve human participants or vertebrate animals. All experimental procedures complied with relevant institutional, national, and international guidelines for environmental research. Field sampling was conducted with the appropriate permissions from Fataca Innovation Hub, in Odemira, in Southern Portugal.

## Abbreviations

The following abbreviations are used in this manuscript:

ANOVA	Analysis of Variance
ATR-FTIR	Attenuated Total Reflectance-Fourier Transform Infrared
COTESI^®^	Companhia de Têxteis Sintéticos S.A.
DEHP	Di(2-ethylhexyl) phthalate
EN	European Norm
MEI	Mean Enzymatic Index
PBAT	Polybutylene adipate terephthalate
PE	Polyethylene
PP	Polypropylene
ROS	Reactive oxygen species
T_base_	Base temperature, 0 °C
W	West

## References

[1] Sintim H.Y., Bary A.I., Hayes D.G., Wadsworth L.C., Anunciado M.B., English M.E., Bandopadhway S., Schaeffer S.M., DeBruyn J.M., Miles C.A. (2020). In situ degradation of biodegradable plastic mulch films in compost and agricultural soils. Sci. Total Environ..

[2] Francioni M., Kishimoto-Mo A.W., Tsuboi S., Hoshino Y.T. (2022). Evaluation of the mulch films biodegradation in soil: A methodological review. Ital. J. Agron..

[3] Zhang H., Miles C., Gerdeman B., LaHue D.G., DeVette L. (2021). Plastic mulch use in perennial fruit cropping systems–A review. Sci. Hortic..

[4] (2018). Plastics-Biodegradable Mulch Films for Use in Agriculture and Horticulture. Requirements and Test Methods.

[5] Tofanelli M.D., Wortman S.E. (2020). Benchmarking the agronomic performance of biodegradable mulches against polyethylene mulch film: A meta-analysis. Agronomy.

[6] Bianchini M., Trozzo L., D’Ottavio P., Giustozzi M., Toderi M., Ledda L., Francioni M. (2022). Soil refinement accelerates in-field degradation rates of soil-biodegradable mulch films. Ital. J. Agron..

[7] Griffin-LaHue D., Ghimire S., Yu Y., Scheenstra E.J., Miles C.A., Flury M. (2022). In-field degradation of soil-biodegradable plastic mulch films in a Mediterranean climate. Sci. Total Environ..

[8] Coutris C., Rivier P.-A., Torp T., Klaus J., Joner E.J. (2025). In situ degradation of biodegradable plastic mulch in Nordic agricultural soils. Chemosphere.

[9] Graf M., Choiselat E., Reay M.K., Bargiela R., Dimitriou A., Liu Q., Elias R.M., Golyshin P.N., Griffiths R., Chadwick D.R. (2025). Biodegradable mulch films exhibit slower-than-expected degradation with negligible effects on soil microbial communities. J. Hazard. Mater..

[10] Senevirathne G.I., Meththa Gimhani T.D., Reay M.K., Perera C., Ariyaratna M., Karunarathna A.K., Chadwick D.R., Jones D.L., Emrah C., Lloyd C.E.M. (2025). In situ degradation of three contrasting plastic mulch films under maize cultivation in tropical conditions. Environ. Adv..

[11] Colachis M., Lilly J.L., Trigg E., Kucharzyk K.H. (2024). Analytical tools to assess polymer biodegradation: A critical review and recommendations. Sci. Total Environ..

[12] Sintim H.Y., Bandopadhyay S., English M.E., Bary A.I., DeBruyn J.M., Schaeffer S.M., Miles C.A., Reganold J.P., Flury M. (2019). Impacts of biodegradable plastic mulches on soil health. Agric. Ecosyt. Evrion..

[13] Machado R.M.A., Bryla D.R., Correia M., Percival D., Polashock J., Retamales J. (2023). Strategies to reduce supraoptimal temperatures in the root zone during field and containerized production of highbush blueberry in warm climates. ISHS Proceedings XII International Vaccinium Symposium, Halifax, NS, Canada, 30 August–1 September 2021.

[14] Campbell G.S., Norman J.M. (1979). An Introduction to Environmental Biophysics.

[15] Wang Z., Li M., Flury M., Schaeffer S.M., Chang Y., Tao Z., Jia Z., Li S., Ding F., Wang J. (2021). Agronomic performance of polyethylene and biodegradable plastic film mulches in a maize cropping system in a humid continental climate. Sci. Total Environ..

[16] Kadera M.A., Sengeb M., Mojid M.A. (2017). Mulching type-induced soil moisture and temperature regimes and water use efficiency of soybean under rain-fed condition in central Japan. Int. Soil Water Conserv. Res..

[17] FAO (2021). Assessment of Agricultural Plastics and Their Sustainability. A Call for Action.

[18] Appiah K.S., Onwona-Agyeman S., Omari R.A., Horiuchi N., Sarkodee-Addo E., Sabi E.B., Fujii Y. (2023). Evaluation of the effectiveness of loose and compressed wood chip mulch in field-grown blueberries-A preliminary study. Agronomy.

[19] Scopetani C., Bellabarba A., Selvolini G., Martellini T., Viti C., Cincinelli A. (2025). Evaluating additive release from conventional and biodegradable mulch films. Sci. Total Environ..

[20] El-Sakhawy M., Kamel S., Salama A., Tohamy H.-A.S. (2018). Preparation and infrared study of cellulose based amphiphilic materials. Cellul. Chem. Technol..

[21] Oliveira T.A., Oliveira R.R., Barbosa R., Azevedo J.B., Alves T.S. (2017). Effect of reprocessing cycles on the degradation of PP/PBAT-thermoplastic starch blends. Carbohydr. Polym..

[22] Tsou C.-H., Chen Z.-J., Yuan S., Ma Z.-L., Wu C.-S., Yang T., Jia C.-F., Guzman M.R.D. (2022). The preparation and performance of poly(butylene adipate) terephthalate/corn stalk composites. Curr. Res. Green. Sustain. Chem..

[23] Jung M.R., Horgen F.D., Orski S.V., Rodriguez V.C., Beers K.L., Balazs G.H., Jones T.T., Work T.M., Brignac K.C., Sarah-Jeanne Royer S.-J. (2018). Validation of ATR FT-IR to identify polymers of plastic marine debris, including those ingested by marine organisms. Mar. Pollut. Bull..

[24] Achimsky L., Audouin L., Verdu J. (1997). Kinetic study of the thermal oxidation of polypropylene. Polym. Degrad. Stab..

[25] Padermshoke A., Kajiwara T., An Y., Takigawa M., Van Nguyen T., Masunaga H., Kobayashi Y., Ito H., Sasaki S., Takahara A. (2022). Characterization of photo-oxidative degradation process of polyolefins containing oxo-biodegradable additives. Polymer.

[26] Anshari R., Tsuboi M., Sato H., Tashiro K., Harumi R., Ozaki Y. (2025). Raman and ATR-FTIR unmask crystallinity changes and carboxylate group and vinyl group accumulation in natural weathering polypropylene microplastic. Sci. Rep..

[27] Thakur S., Mathur S., Saraf M., Patel S., Jadav R., Menon S. (2025). Bacterial isolation and evaluation for degradation of unpretreated LDPE from waste dump soils. J. Pure Appl. Microbiol..

[28] da Silva D.J., Wiebeck H. (2022). ATR-FTIR Spectroscopy Combined with Chemometric Methods for the Classification of Polyethylene Residues Containing Different Contaminants. J. Polym. Environ..

[29] Di Pippo F., Bocci V., Amalfitano S., Crognale S., Levantesi C., Pietrelli L., Di Lisio V., Martinelli A., Rossetti S. (2023). Microbial colonization patterns and biodegradation of petrochemical and biodegradable plastics in lake waters: Insights from a field experiment. Front. Microbiol..

[30] Koseki H.C.M., Bergozza M., Braz C.J.F., Alves T.S., Barbosa R. (2025). Polypropylene reinforced with hollow glass microspheres: Effect of thermal aging and reprocessing. Polímeros Ciência Tecnol..

[31] Sonohata H., Manago G., Seike S., Kitawaki H., Tanabe T. (2025). Evaluation of plastic waste degradation using terahertz spectroscopy for material recycling. Recycling.

[32] Salomez M., George M., Fabre P., Touchaleaume F., Cesar G., Lajarrige A., Gastaldi E. (2019). A comparative study of degradation mechanisms of PHBV and PBSA under laboratory-scale composting conditions. Polym. Degrad. Stab..

[33] Pedra F., Inácio M.L., Fareleira P., Oliveira P., Pereira P., Carranca C. (2024). Long—Term effects of plastic mulch in a sandy—Loam soil cultivated with blueberry at southern Portugal. Pollutants.

[34] (2018). Textiles—Determination of Resistance to Water Penetration—Hydrostatic Pressure Test.

[35] (2013). Textiles—Tensile Properties of Fabrics, Part 1: Determination of Maximum Force and Elongation at Maximum Force Using the Strip Method.

[36] Li X., Wang Q., Jiang N., Lv H., Liang C., Yang H., Yao X., Wang J. (2023). Occurrence, source, ecological risk, and mitigation of phthalates (PAEs) in agricultural soils and the environment: A review. Environ. Res..

[37] Pedra F., Oliveira P., Carranca C. (2025). Monitorização Das Condições do Solo em Cultivo Sob Diferentes Coberturas.

[38] Elnesr M.N., Alazba A.A. (2016). An integral model to calculate the growing degree-days and heat units, a spreadsheet application. Comput. Electron. Agric..

[39] Bocock K.L., Gilbert O., Capstick C.K., Twinn D.C., Waid J.S., Woodman M.J. (1960). Changes in leaf litter when placed on the surface of soils with contrasting humus types I. Losses in dry weight of oak and ash leaf litter. Eur. J. Soil Sci..

[40] Neto C., Carranca C., Clemente J. (2009). Senescent leaf decomposition in a Mediterranean pear orchard. Eur. J. Agron..

[41] StatSoft (2013). StatSoft Incorporated. Electronic Statistics Textbook.

